# Supplementary material for: CREB1 Is Involved in miR-134-5p-Mediated Endometrial Stromal Cell Proliferation, Apoptosis, and Autophagy
Source: Cells. 2023 Oct 31;12(21):2554. doi: 10.3390/cells12212554 (PMC10649013; doi:10.3390/cells12212554)
Supplement: Supplementary file 1 [file cells-12-02554-s001.zip › Supplementary figures.pdf]

Figure S1

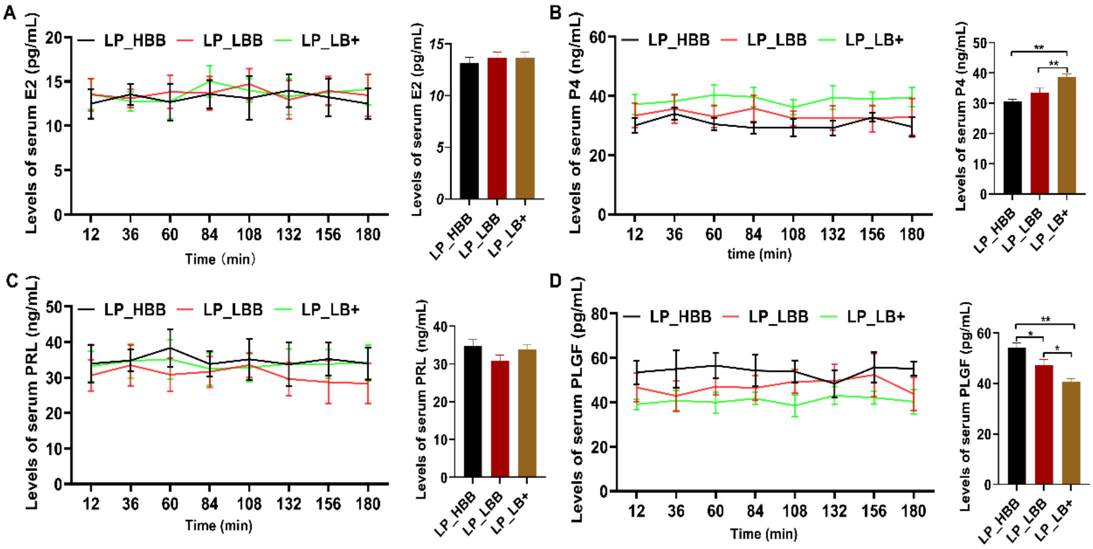

Figure S1 Detection of serum hormone and growth factor content in different groups of sheep. (A) Analysis of serum estrogen (E2) levels. (B) Analysis of serum progesterone (P4) levels. (C) Analysis of serum prolactin (PRL) levels. (D) Analysis of serum placental growth factor (PLGF) levels. Results were expressed as mean ± SEM. *$p < 0.05$, **$p < 0.01$.

Figure S2

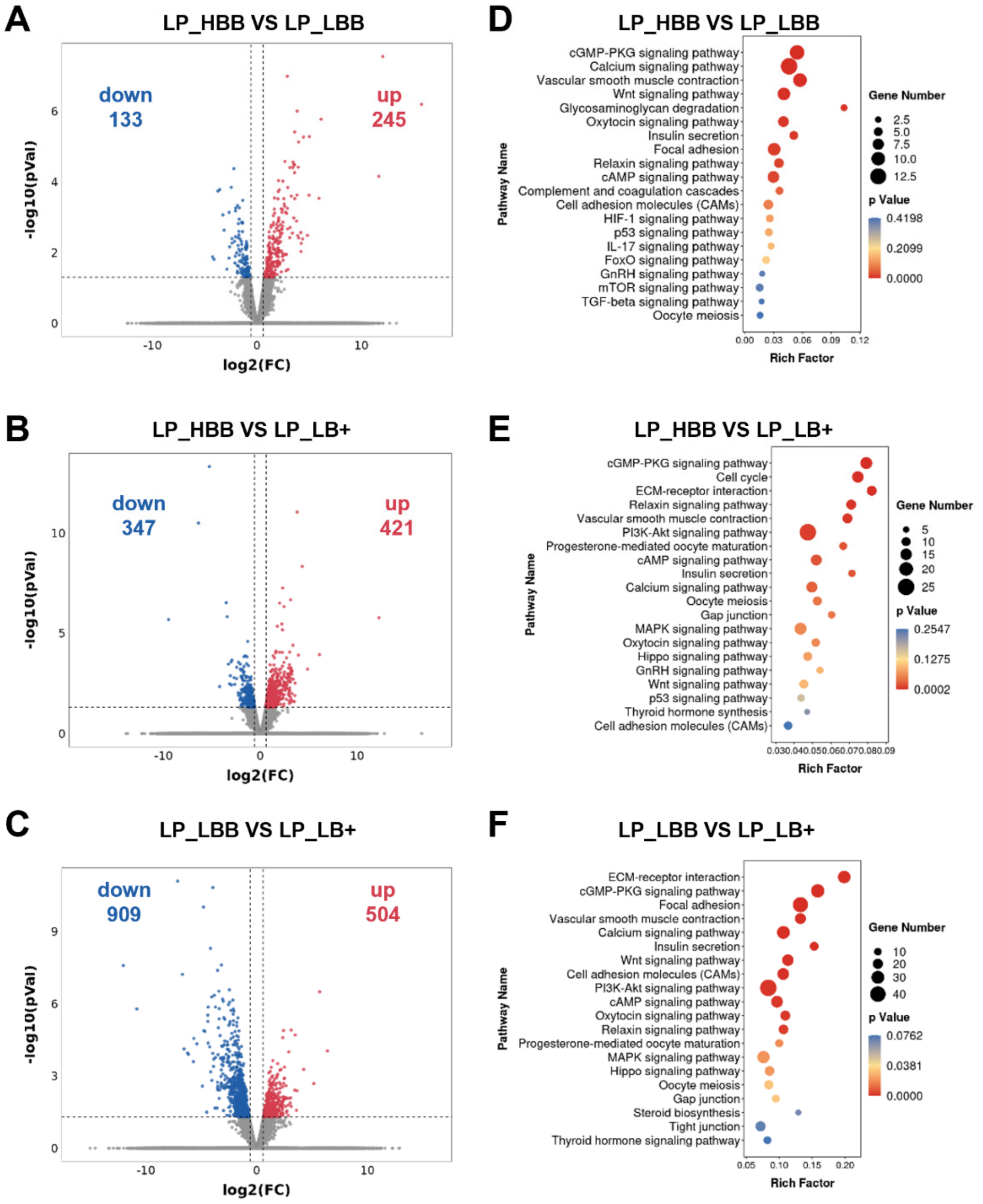

Figure S2 Functional annotation and analysis of endometrial differentially expressed genes (DEGs) in luteal phase of different fecundity sheep. (A-C) The volcano plot of DEGs among different groups. (D-F) The KEGG enrichment scatterplot of DEGs among different groups.

Figure S3

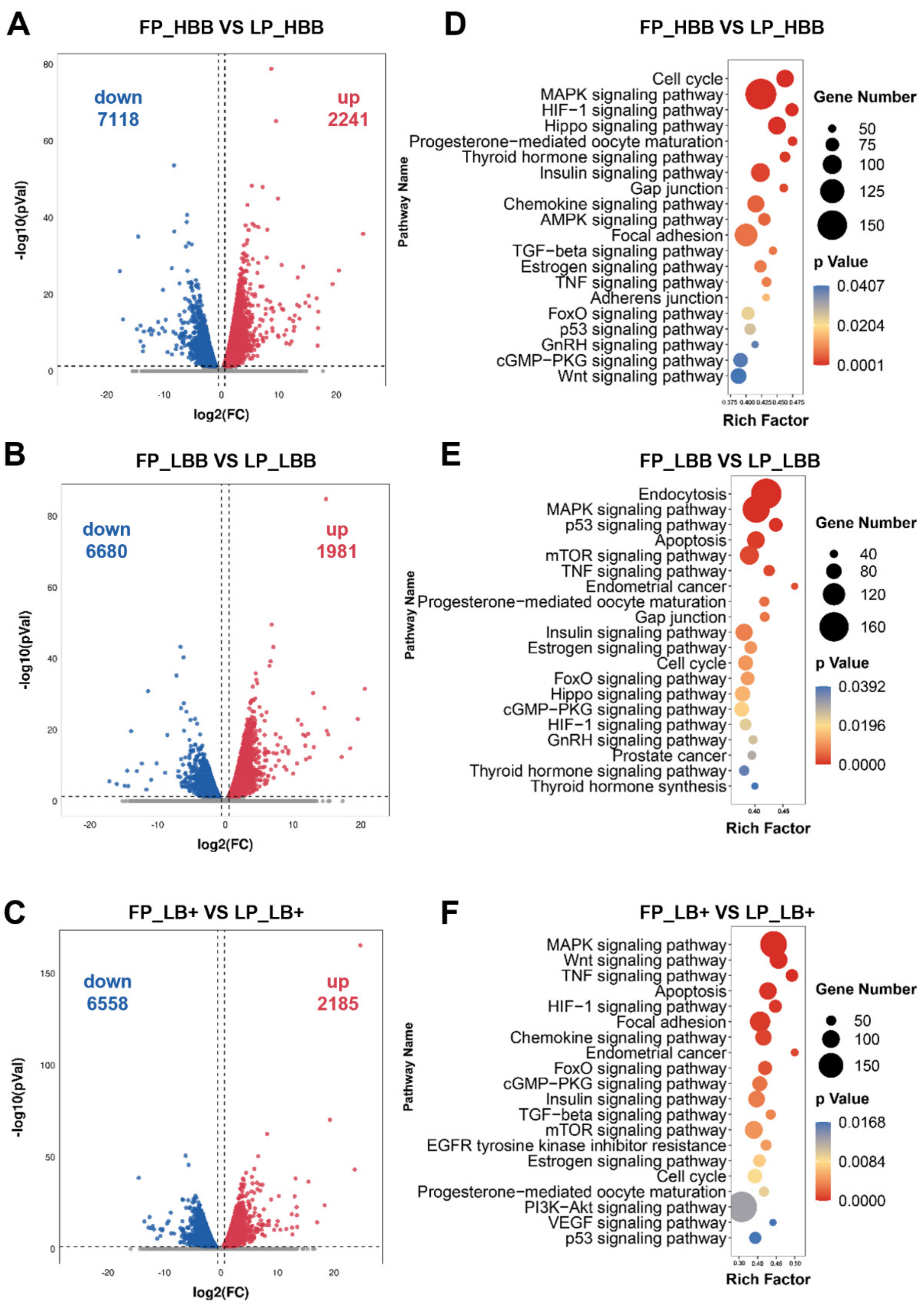

Figure S3 Functional annotation and analysis of endometrial differentially expressed genes (DEGs) in follicle-luteal transformation of different fecundity sheep. (A-C) The volcano plot of DEGs among different groups. (D-F) The KEGG enrichment scatterplot of DEGs among different groups.

Figure S4

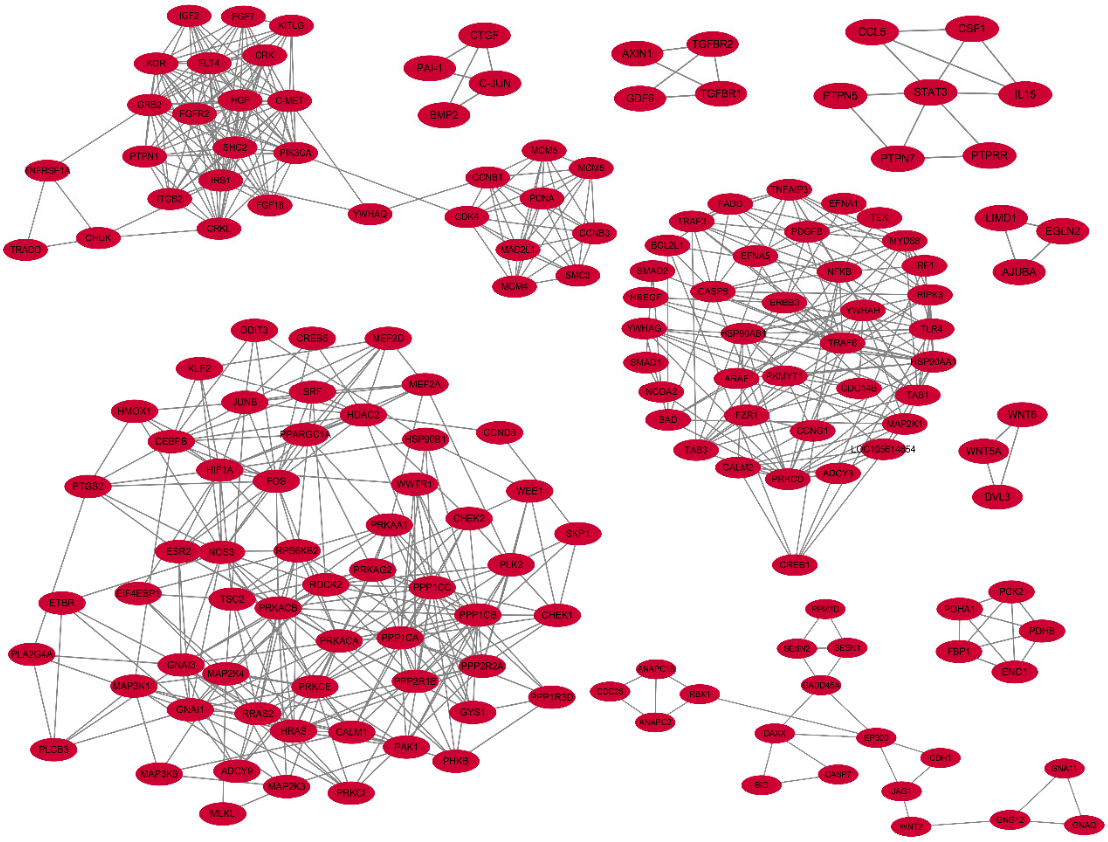

Figure S4 Protein-Protein Interaction Networks (PPI) analysis of differential expressed mRNAs related to female reproduction of endometrial of Hu sheep with high- and low-fertility in follicle-luteum transformation process.

Figure S5

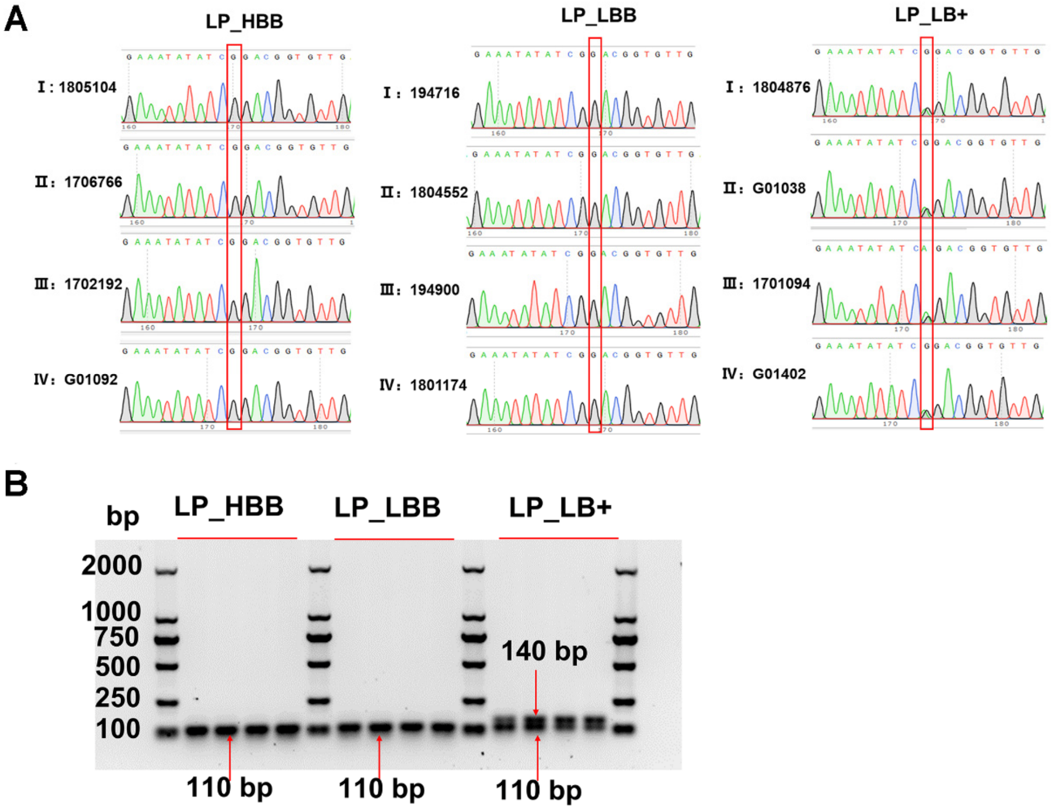

Figure S5 Detection of *FecB* genotype in Hu sheep with different fecundity. (A and B): FecB genotype was examined by PCR-SSCP (A) and PCR-RFLP (B). The red box represents mutation site (A→G). The wild-type allele (+) is 140-bp, and the mutant allele is 110-bp. and. HBB, high prolificacy group carrying the $FecB^{BB}$ genotype; LBB, low prolificacy group carrying the $FecB^{BB}$ genotype; LB+, low prolificacy group carrying the $FecB^{B+}$ genotype; LP, luteal phase.

Figure S6

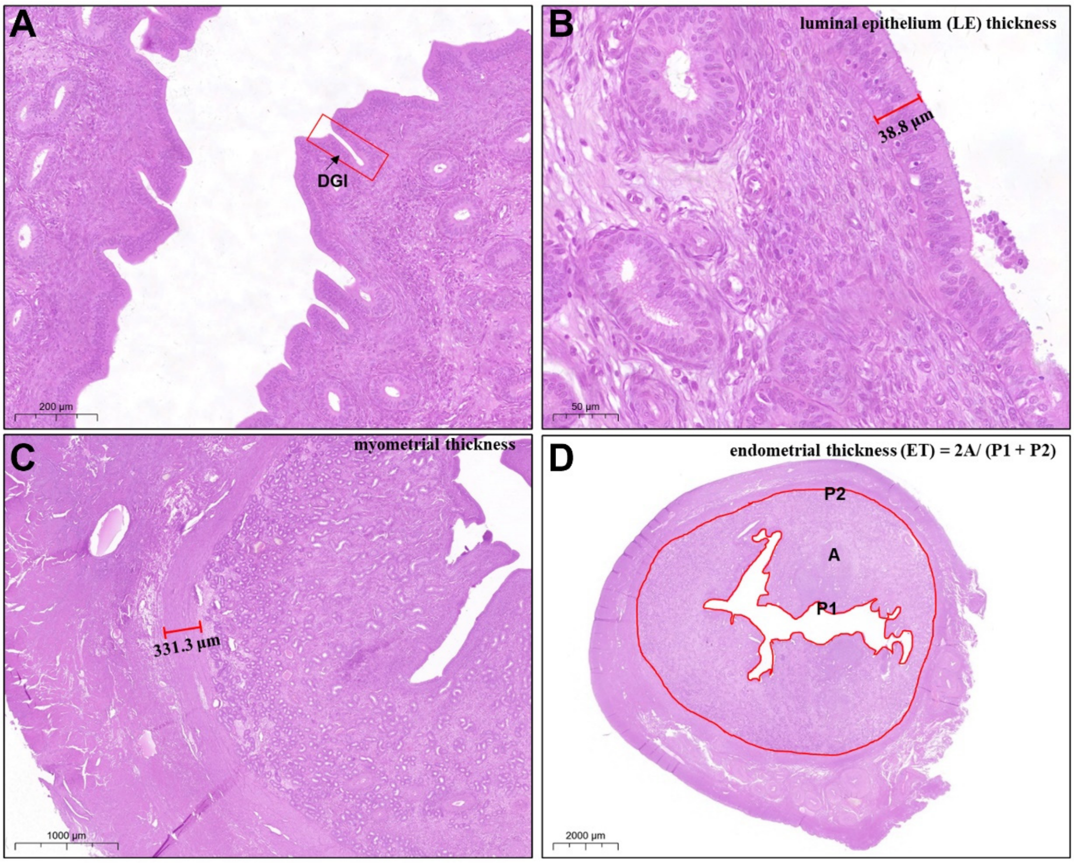

Figure S6 Schematic diagrams of Hu sheep uterine morphology analysis. (A) Schematic diagram of endometrial ductal gland invaginations (DGI) analysis. (B) Schematic diagram of luminal epithelium (LE) thickness analysis. (C) Schematic diagram of myometrial thickness analysis. (D) Schematic diagram of endometrial thickness (ET) analysis. ET = 2A/ (P1 + P2): the calculated area is the total area (A) of the endometrium on a uterine cross-section; the calculated inner perimeter (P1) is measured by tracing the lumen and the endometrium-myometrium interface perimeter (P2) is measured by tracing this interface.

Figure S7

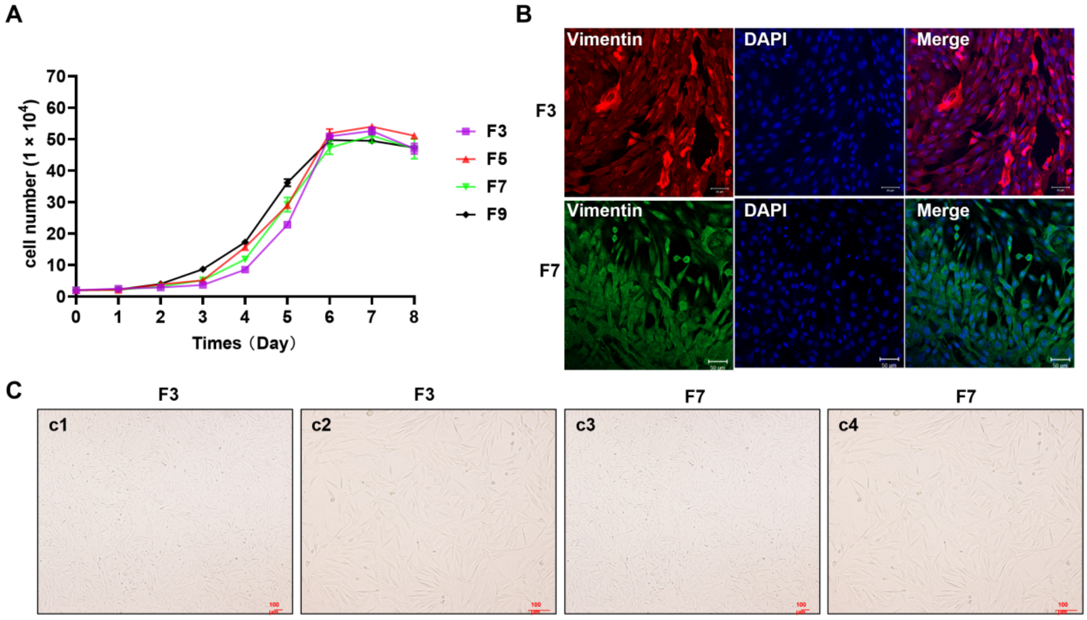

Figure S7 Information about the passage number or validation of genotypic and phenotypic stability. (A) The cell growth curve of endometrial stromal cells (ESCs). (B) Identification of ESCs. Blue: DAPI-stained cell nuclei; Red or green: fluorescence staining of vimentin protein. Scale bar = 50 μM. (C) Cell morphology of F3 (c1 and c2) and F7 ESCs (c3 and c4). Scale bar = 100 μM.
